# Progestin-related breast volume changes in a woman with complete androgen insensitivity syndrome (CAIS)

**DOI:** 10.1530/EDM-22-0346

**Published:** 2023-05-08

**Authors:** Benthe A M Dijkman, Christel J M de Blok, Koen M A Dreijerink, Martin den Heijer

**Affiliations:** 1 Department of Endocrinology and Metabolism, Amsterdam UMC location Vrije Universiteit Amsterdam, De Boelelaan 1117, Amsterdam, the Netherlands

**Keywords:** Adult, Female, White, Netherlands, Adipose tissue, Puberty, Unusual effects of medical treatment, May, 2023

## Abstract

**Summary:**

A 31-year-old woman with complete androgen insensitivity syndrome (CAIS) experienced breast volume fluctuations during biphasic hormone replacement therapy consisting of estradiol and cyclical dydrogesterone, a progestin. 3D breast volume measurements showed a 100 cc volume (17%) difference between estradiol monotherapy and combined estradiol and dydrogesterone treatment. Progestogen-dependent breast volume changes have not been reported in the literature. Our findings suggest a correlation between progestogen use and breast volume. Due to the rapid cyclical changes, we hypothesize that the effect is caused by fluid retention.

**Learning points:**

- There is limited reports available on the effects of progesterone on breast development and volume.
- 3D imaging provides an easy-to-use method to quantify breast volume.
- The patient in our case description clearly showed that cyclic progesterone use might induce substantial cyclic changes in breast volume.
- In women with complete androgen insensitivity syndrome (CAIS), monotherapy with estrogen or continuous supplementation of progesterone might be preferable over cyclic progesterone use.

## Background

Estradiol is known to be the main initiator of breast development and growth during puberty. Breast maturation following estradiol exposure includes ductal proliferation and accumulation of adipose tissue. Progesterone is known for its proliferative effects on mammary tissue in women, in particular lobulo-alveolar maturation (1, 2).

Among the disorders of sex development (DSD), androgen insensitivity syndrome (AIS) is the most frequent condition in 46, XY phenotypic women with an estimated prevalence of 6.4 per 100,000 females (3, 4, 5). The most common type is complete AIS (CAIS), which is characterized by a phenotype with typical female external genitalia and undescended testes (4, 6). After gonadectomy, life-long hormone replacement therapy is necessary for full pubertal development and maintenance of bone mineralization (4, 7, 8, 9). The most common recommendation for hormone replacement therapy is estrogen monotherapy, and to date, no evidence has been found that the addition of (cyclical) progestogens may benefit well-being in women who do not have a uterus (8, 9, 10, 11). However, as illustrated by this case, in clinical practice, women with CAIS occasionally receive a combination therapy instead of estrogen monotherapy.

During the menstrual cycle, estradiol and progesterone are believed to be involved in the cyclical changes women experience in their breasts, including mastalgia and tenderness. However, progesterone is not typically associated with significant breast volume changes. In this case report, we present potential progestin-related breast volume changes in a woman with CAIS who used biphasic hormone replacement.

## Case presentation

A 31-year-old woman with CAIS visited our endocrinology department for counseling regarding hormone replacement therapy. At the age of 16, she was analyzed at the endocrinology department of another hospital because of primary amenorrhea. Genetic testing at that time showed a 46XY karyotype. Testosterone was 19.2 nmol/L, estradiol was 80 pmol/L, luteinizing hormone (LH) was 35 U/L, and follicle-stimulating hormone (FSH) was 24 U/L. Magnetic resonance imaging (MRI) showed gonadal structures adherent to both inguinal canals and absence of the proximal vagina, uterus, and adnexa. Based on these data the diagnosis of CAIS was made (genotyping of androgen receptor gene was not performed). Intra-abdominal testes were surgically removed and showed immature testis tissue. Subsequently, she started with hormone replacement therapy in the form of biphasic estradiol/dydrogesterone 2/10 mg (days 1–14: estradiol 2 mg, days 15–28: estradiol 2 mg/dydrogesterone 10 mg, all daily doses). We saw her several years later. She had noticed mastalgia and breast volume fluctuations of about one cup size difference during her hormone replacement therapy cycles. Vital parameters showed a blood pressure of 118/71 mmHg, height of 180 cm, weight of 62 kg, and body mass index of 19 kg/m^2^. Blood tests and dual-energy x-ray absorptiometry (DXA) imaging were performed during the visit. The serum concentration of 17-beta-estradiol was 397 pmol/L, testosterone 0.5 nmol/L, LH 19 U/ L, and FSH 32 U/L. The DXA scan was normal (lumbar spine L1–L4 = 1.01, T-score = −0.3; bone density left hip = 0.85, T-score = −0.8; bone density femoral neck = 0.82, T-score = −0.3). At first, we switched from femoston 2/10 (cyclic) to estradiol 3 mg daily monotherapy. This stopped the fluctuations in breast volume. To check whether these fluctuations indeed were a result of progesterone, we did a trial with the addition of progestin 10 mg continuously, keeping the estradiol dose at 3 mg daily. The breast volume measurements were performed while she was stable on estradiol 3 mg for 6 months.

## Treatment

As we reasoned there was no need for progestogen because of the absence of a uterus, the hormone replacement therapy was changed to estradiol valerate monotherapy at a dose of 3 mg daily.

## Outcome and follow-up

During a follow-up appointment, the patient had also noticed that the breast volume fluctuations had stopped, but she also experienced an overall decrease in breast volume. She chose to continue the combination of estradiol and continuous progestin therapy (dydrogesterone 10 mg daily). In addition to a positive effect on breast volume, the patient experienced an improvement in overall well-being.

## Investigation

In order to measure the breast volume fluctuations, we made breast 3D images (using a VECTRA 3D scanner with software version 5.7.1. as described in Blok *et al.* (12)). This method has high accuracy and low inter- and intra- observer variation (13). The first 3D image was taken while she was on estradiol monotherapy (3 mg daily) for 6 months, and the second image 4 weeks after the addition of dydrogesterone continuously (10 mg daily). The breast volume estimations performed on the 3D images showed a right breast volume of 673 cc and a left breast volume of 758 cc during estradiol monotherapy. Four weeks after initiation of the combination treatment, a breast volume of 784 cc was found in the right breast. The left breast had a volume of 875 cc. Thus, the combination treatment resulted in a 111 cc (17%) breast volume increase in the right breast and a 117 cc (16%) increase in the left breast.

## Discussion

We present a case of a 31-year-old woman with CAIS with breast volume fluctuations of more than 100 cc between estradiol monotherapy and combination therapy of estradiol and dydrogesterone. In the literature, the influence of progesterone on breast growth and breast volume has been debated; however, no clinical trials have been conducted (14).

When choosing the type of hormone replacement therapy, administration of progestogen in combination with estradiol is advised in women with a uterus (e.g. postmenopausal women and women with DSD who have a uterus) because long-term use of conjugated estrogens alone leads to spotting and increases the risk of adenocarcinoma of the endometrium (15). Estrogen administration alone is the recommendation for (postmenopausal) women who do not have a uterus (e.g. women who underwent hysterectomy, women with CAIS) (8, 9, 10, 11). A downside of using preparations that are designed for post-menopausal women is that the estradiol dose might be relatively low, as was in the case we presented. Although bone density was in the normal range, women with low estradiol levels might be at risk to develop osteoporosis during their life course.

As the cyclical changes in the breast volume of our patient (±100 cc/16–17%) correspond to almost a bra-cup size difference, the observation seems significant. MRI studies on breast volume change during the menstrual cycles showed volume changes of 76 mL (13.6%) (16). For example, the increase is similar to the average overall increase in breast volume in transgender women, which is approximately 72 cc after up to 3 years of use of gender-affirming hormone therapy (12). In the past, studies addressing the influence of sex hormones on fluid status have demonstrated that progestogens cause sodium and fluid retention due to activation of the renin–angiotensin–aldosterone system, which could explain the rapid breast volume changes in this patient (17, 18). The mastalgia our patient experienced when she used estradiol/dydrogesterone 2/10 may also be related to this mechanism. Androgens have an inhibitory role in breast development (19). Although the observation of the breast volume changes in this case when using the progestin is likely to be unrelated to the specific diagnosis of CAIS, one could speculate that the complete absence of breast growth inhibition by androgens on the breast tissue of this patient could have played a role in the observed effect. Finally, this case description illustrates the advantage of 3D breast imaging to evaluate subjective changes in breast volume and to quantify the effect of hormonal therapy.

## Declaration of interest

There are no conflicts of interest.

## Funding

This study did not receive any specific grant from any funding agency in the public, commercial or not-for-profit sector.

## Patient consent

Written informed consent for publication of their clinical details was obtained from the patient.

## Patient’s perspective

I appreciate very much that I was enabled to objectively confirm my experience that my breast size was influenced significantly during my monthly therapy cycle. I hope this finding will promote more research on this topic so that it can ultimately help other people using hormone therapy.

## Author contribution statement

B Dijkman and C de Blok did the 3D measurements of breast volume and prepared a draft manuscript. K Dreijerink and M den Heijer supervised the writing. M den Heijer is the treating physician of the patient.
